# Effects of change in walking speed on time-distance parameters in post-stroke hemiplegic gait

**DOI:** 10.20407/fmj.2021-016

**Published:** 2022-01-25

**Authors:** Ken Tomida, Kei Ohtsuka, Toshio Teranishi, Hiroki Ogawa, Misaki Takai, Akira Suzuki, Kenji Kawakami, Shigeru Sonoda

**Affiliations:** 1 Graduate School of Health Sciences, Fujita Health University, Toyoake, Aichi, Japan; 2 Fujita Health University Nanakuri Memorial Hospital, Tsu, Mie, Japan; 3 Faculty of Rehabilitation, School of Health Sciences, Fujita Health University, Toyoake, Aichi, Japan; 4 Department of Rehabilitation Medicine II, School of Medicine, Fujita Health University, Tsu, Mie, Japan

**Keywords:** Stroke, Time-distance parameters, Walking speed

## Abstract

**Objectives:**

In stroke patients, the assessment of gait ability over time is important. For quantitative gait assessment using measuring devices, the walking speed condition for measurement is generally based on the patient’s preferred walking speed or the maximum walking speed at the time of measurement. However, because walking speed often increases during the convalescent stage, understanding the effects of change in walking speed on gait when comparing the course of recovery is necessary. Although several previous studies have reported the effects of change in walking speed on gait in stroke patients, the time-distance parameters described in these reports may not be generalizable because of the small case numbers. Therefore, we measured treadmill gait at the preferred walking speed (PWS) and 1.3 times the PWS (130% PWS) in 43 post-stroke hemiplegic patients and analyzed the effects of change in walking speed on time-distance parameters.

**Methods:**

Forty-three patients with hemiplegia after a first stroke, who were able to walk on a treadmill under supervision, were recruited as subjects. Using a three-dimensional motion analysis system, treadmill gait was assessed under two conditions: PWS and 130% PWS. The primary outcome measures were the time-distance parameters, which were compared between the PWS and 130% PWS conditions.

**Results:**

Cadence, stride length, and step length of the affected and unaffected lower limbs increased significantly at 130% PWS compared with at PWS. In terms of actual time, single stance time and initial and terminal double stance time in both affected and unaffected limbs decreased significantly at 130% PWS. In terms of relative time (% of the gait cycle), compared with PWS, relative single stance time increased significantly, whereas relative initial and terminal double stance times decreased significantly at 130% PWS in both the affected and unaffected limbs.

**Conclusions:**

This study on treadmill gait in patients with hemiplegia after a first stroke confirmed the effects of change in walking speed on time-distance parameters. Our results will help in the interpretation of time-distance parameters measured under different walking speed conditions.

## Introduction

Improving the gait ability of post-stroke hemiplegic patients is an important rehabilitation goal of stroke patients. To evaluate changes in gait ability over time, which reflect the effect of rehabilitation, an appropriate method for assessing gait ability is required. Conventionally, gait ability is evaluated by determining the degree of gait independence using tools, such as the functional ambulation categories,^1^ Functional Independence Measure (FIM),^2^ and Gait Ability Assessment for hemiplegics (GAA),^3^ or walking speed by measuring the time to walk 5 or 10 m.^4^ Three-dimensional motion analyzer and ground reaction force measurement devices are also used for kinematic and kinetic evaluations.^5,6^ These measurement devices are effective evaluation tools because they provide quantitative evaluations of various parameters of gait, which include time-distance parameters, joint angles, and joint moments.

When gait is assessed using these measurement devices, the walking speed condition is typically set according to the preferred walking speed (PWS) or the maximum walking speed at the time of measurement. However, for post-stroke hemiplegic patients in the convalescent stage, walking speed increases during the course of convalescence,^7^ and the walking speed associated with walking conditions, such as comfort, can differ between the first and second tests. Therefore, when interpreting assessment results, it is imperative to understand the effects of change in walking speed based on the time of measurement.

Numerous studies have investigated the effects of change in walking speed on gait time-distance parameters in healthy subjects.^8–13^ For post-stroke hemiplegic patients, one study recruited a large number of patients and compared time-distance parameters according to walking speed with those of healthy subjects matched for baseline data.^14^ However, few reports have examined the impact of change in walking speed per se on gait time-distance parameters in post-stroke hemiplegic patients. A review of previous studies in post-stroke hemiplegic patients identified reports on the effects of walking speed on time-distance parameters,^15–17^ joint angles,^15,16^ and muscle activation.^17^ Studies that focused on time-distance parameters that are easy to use clinically include small-scale studies^16,17^ and an investigation of the effects of change in walking speed on distance parameters, such as stride length and step length, as well as typical time parameters, such as cadence.^15^ However, few studies subdivided time-distance parameters and analyzed them comprehensively.

Based on these studies, we studied 43 post-stroke hemiplegic patients by measuring treadmill gait during two speed conditions: PWS and 1.3 times the PWS. We analyzed the effects of change in walking speed on time-distance parameters. Moreover, we performed a comprehensive analysis of cadence and actual and relative time parameters.

## Methods

### Study design

The study design was a retrospective cohort study.

### Subjects

Among patients with hemiplegia after a first stroke who were admitted to the comprehensive inpatient rehabilitation wards of our hospital, we recruited 43 patients who were able to walk on a treadmill, under supervision and wearing an ankle-foot orthosis, 6 weeks after admission. Patient baseline data were acquired, which included age, sex, affected side, duration after onset, FIM-walk score,^2^ and stroke impairment assessment set total lower extremity motor score (SIAS-L/E)^18^ (Table 1). This study was approved by the Medical Research Ethics Review Committee of Fujita Health University (HM19-254).

### Measurements

Treadmill gait measurement was conducted using Kinema Tracer^®^ (Kissei Comtec Co., Ltd., Matsumoto, Japan), a three-dimensional motion analysis system. All subjects used a handrail. The setting for orthosis was that used in gait training during physiotherapy. The walking speeds for the measurement were level ground PWS and 1.3 times the level ground PWS (130% PWS). A safety harness was used to prevent falls, without body weight support.

Treadmill gait measurement was conducted according to the method described in a previous study.^5^ Markers were attached to 10 locations on the left and right acromia, hip joints, knee joints, lateral malleoli, and fifth metatarsal heads. The sampling frequency was 60 Hz, and the measurement time was 20 seconds.

To determine each subject’s PWS, which is the basis of the measurement speed, the 10-m walking time on level ground at the subject’s preferred speed was measured three times, and the average value was defined as the PWS. In addition, before the treadmill gait measurement, subjects practiced until they were adequately accustomed to treadmill walking.

### Outcome measures

The primary outcome measures were time-distance parameters obtained from the treadmill gait measurement. These parameters comprised walking speed, stride length, cadence, step length of the affected limb (step length-A), step length of the unaffected limb (step length-U), single stance time of the affected limb (SST-A), single stance time of the unaffected limb (SST-U), terminal double stance time (TDST; defined as the time of double-limb support when the weight is unloaded from the affected limb), initial double stance time (IDST, defined as the time of double-limb support when the weight is loaded onto the affected limb), single stance time of the affected limb as a percentage of the gait cycle (SSTP-A), single stance time of the unaffected limb as a percentage of the gait cycle (SSTP-U), terminal double stance time as a percentage of the gait cycle (TDSTP), and initial double stance time as a percentage of the gait cycle (IDSTP).

### Statistical analysis

Statistical analyses were performed using SPSS Statistics 19 (International Business Machines Corp., Armonk, NY, USA). Paired t-tests were used to compare the time-distance parameters between the two walking speed conditions. A *p*-value less than 0.05 was considered significant.

## Results

**Figure 1:**
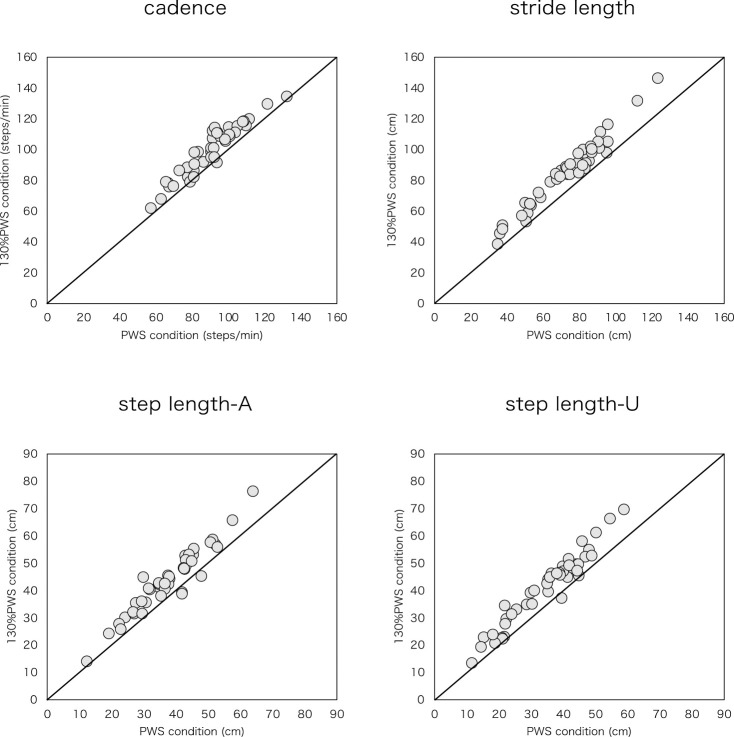
Scatter plot of distance parameters and cadence for PWS condition versus 130% PWS condition

**Figure 2:**
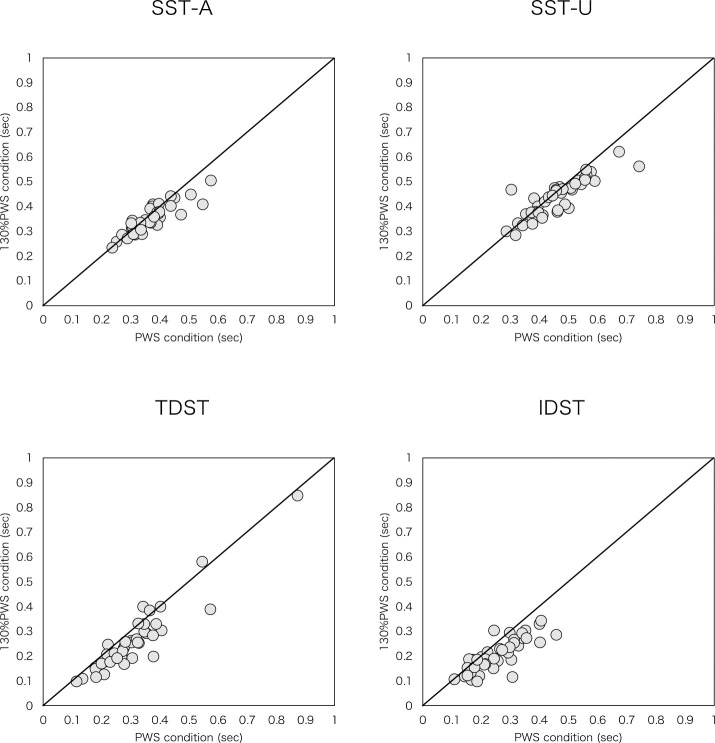
Scatter plot of actual time parameters for PWS condition versus 130% PWS condition

**Figure 3:**
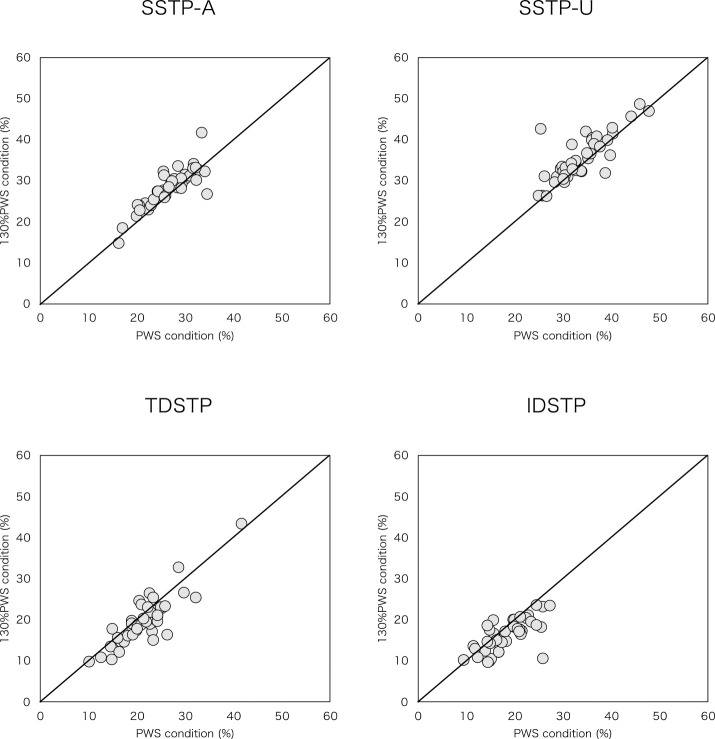
Scatter plot of relative time parameters (as a percentage of the gait cycle) for PWS condition versus 130% PWS condition

Walking speeds were 2.0±0.7 km/h in the PWS trial and 2.6±0.9 km/h in the 130% PWS trial. Cadence and distance parameters, comprising stride length, step length-A, and step length-U, increased significantly at 130% PWS compared with at PWS (Table 2, Figure 1). Regarding the time parameters, actual time parameters of SST-A, SST-U, TDST, and IDST decreased significantly at 130% PWS than at PWS (Table 2, Figure 2). Relative time parameters of SSTP-A and SSTP-U increased significantly at 130% PWS than at PWS, whereas TDSTP and IDSTP decreased significantly at 130% PWS than at PWS (Table 2, Figure 3).

Table 3 shows the number of subjects who showed an increase, decrease, or no change for each time-distance parameter at 130% PWS compared with PWS. The percentage of subjects showing results different from the overall trend was less than 30% for cadence, stride length, step length-A, step length-U, TDST, IDST, SSTP-A, SSTP-U, TDSTP, and IDSTP; however, the percentage tended to be slightly higher for SST-A (32.6%) and SST-U (34.9%) (Table 3).

## Discussion

In this study, we investigated the changes in time-distance parameters associated with changes in walking speed in post-stroke hemiplegic patients. With increased walking speed, all distance parameters increased significantly, whereas all actual time parameters decreased significantly. For relative time parameters, SSTP-A and SSTP-U increased significantly, whereas TDSTP and IDSTP decreased significantly. Cadence increased significantly.

Murray et al.^8^ analyzed gait at free speed and fast speed in healthy subjects. For time-distance parameters, they reported decreases in the times of gait cycle, stance phase, swing phase, double-limb support, stance phase as a percentage of the gait cycle, and double-limb support as a percentage of the gait cycle as well as increases in the time of swing phase as a percentage of the walking cycle, stride length, and cadence during fast speed walking compared with that during free speed walking. Liu et al.^12^ also reported that as walking speed increased, the times of gait cycle, stance phase as a percentage of the gait cycle, and double-limb support as a percentage of the gait cycle decreased, whereas the time of swing phase as a percentage of the gait cycle, stride length, and cadence increased.

Tyrell et al.^16^ analyzed the effects of increasing treadmill walking speed on gait pattern in 20 stroke patients with respect to time-distance and kinematic parameters. For time-distance parameters, step lengths and single-limb support time as a percentage of the gait cycle in the affected and unaffected limbs increased, whereas the double-limb support time as a percentage of the gait cycle decreased, as walking speed increased. Lamontagne et al.^17^ reported similar results in 12 stroke patients. An overview of these previous studies has revealed that the effects of speed increase on gait time-distance parameters are similar in healthy subjects and post-stroke hemiplegic patients, although stroke patients have large inter-individual variability.^19^ Tyrell et al.^16^ noted small sample size as a limitation of their study. Our study analyzed 43 post-stroke hemiplegic patients, and results showed trends similar to previous studies. Therefore, our results may be generalized across stroke patients even to those with large individual variations.

It is well established that an increase in walking speed prompts a necessary increase in cadence and stride length,^8,9,12,13,15^ and an increase in cadence is synonymous with a decrease in the time of one gait cycle. The reduction in actual time of each phase observed in post-stroke hemiplegic patients is considered a temporal strategy in response to a speed increase.

However, in terms of relative time (as a percentage of the gait cycle), the relative single stance time increased while the relative double stance time decreased, in both the affected and unaffected limbs. Murray et al.^8^ analyzed the effect of increased speed on gait in healthy subjects. In their discussion on the time parameters, they proposed that the amount of decrease in swing time is less than that in other phases because swinging the lower limb forward through a greater distance in a shorter time is necessary to achieve the longer step length needed for faster walking. In addition, the generation of propulsive force and rapid forward swing of the lower limbs by increasing the moment of push-off (ankle plantar flexion) in the late stance phase and pull-off (hip joint flexion) in the initial swing phase and increasing angular velocity are important factors for increasing walking speed in stroke patients.^20,21^ Moreover, the smooth push-off and pull-off likely support the decrease in double stance time. In other words, we considered that by increasing the step length to allow a rapid swing-out and propulsion in response to a speed increase, the ratio of decrease in swing time was less than that in the double stance time.

When we examined the results of individual subjects, we observed a slightly high percentage of subjects whose single stance time in both the affected and unaffected limbs deviated from the overall trend of all subjects. Beaman et al.^22^ reported that the strategies used in response to a speed increase are not uniform among subjects. Furthermore, because stroke patients have considerable inter-individual variability,^19^ individual differences in functions and response strategies in the affected or unaffected limb likely contribute to the observed finding. Clarifying the cause of such atypical trends in response to a speed increase may be valuable for improving the evaluation of gait ability and designing gait training programs. Thus, this aspect warrants further investigation.

While performing gait evaluation using a measurement device, the walking speed condition is often set according to the patient’s PWS or their maximum walking speed at the time of measurement. Our results will help in the interpretation of time-distance parameters measured under different speed conditions.

## Study limitations

In this study, a handrail and orthosis were used during measurement. The use of a handrail and orthosis may influence the change in walking speed and time-distance parameters, and therefore, it is a confounding factor of this study.

## Conclusion

Treadmill gait measurement was performed using a three-dimensional motion analyzer under two speed conditions (PWS and 130% PWS) in 43 patients with hemiplegia after a first stroke. Subjects were permitted use of orthosis and a handrail, and measurements were conducted using a safety harness to prevent falls, without body weight support. The orthosis setting was that used for gait training during physiotherapy. The primary outcome measures were time-distance parameters, and these were compared between the two conditions of PWS and 130% PWS.

Cadence, stride length, step length-A, and step length-U increased significantly at 130% PWS compared with at PWS. Among the time parameters, actual SST-A, SST-U, TDST, and IDST decreased significantly at 130% PWS. Relative (as a percentage of the gait cycle) SSTP-A and SSTP-U increased significantly, whereas relative TDSTP and IDSTP decreased significantly at 130% PWS. These trends were similar to those reported in previous studies in healthy subjects. However, certain subjects showed SST-A and SST-U results that diverged from the overall trend.

## Figures and Tables

**Table1:** Baseline data of patients

Number of subjects	(n)	43
Age	(years)	60.7±12.5
Sex (male/female)	(n)	30/13
Affected side (right/left)	(n)	24/19
Duration after onset	(days)	78.0±14.9
SIAS-L/E	(points)	7.8±2.2
FIM-walk	(points)	5.5±0.5

Abbreviations: FIM-walk, Functional Independence Measure-walk score; SIAS-L/E, Stroke Impairment Assessment Set-total lower extremity motor score.

**Table2:** Time-distance parameters at PWS condition and 130% PWS condition

Parameter	Unit	PWS condition	130% PWS condition	P-value
walking speed	(km/h)	2.0±0.7	2.6±0.9	<.01
cadence	(steps/min)	89.9±16.1	99.2±16.8	<.01
stride length	(cm)	72.4±20.2	85.0±22.4	<.01
step length-A	(cm)	37.8±10.6	43.8±11.6	<.01
step length-U	(cm)	34.6±11.7	41.1±13.0	<.01
SST-A	(sec)	0.36±0.07	0.35±0.06	<.01
SST-U	(sec)	0.46±0.10	0.44±0.08	<.01
TDST	(sec)	0.31±0.13	0.26±0.13	<.01
IDST	(sec)	0.25±0.08	0.21±0.07	<.01
SSTP-A	(%)	26.7±4.5	28.2±4.6	<.01
SSTP-U	(%)	33.5±5.3	35.4±5.4	<.01
TDSTP	(%)	21.6±5.4	20.0±6.0	<.01
IDSTP	(%)	18.2±4.4	16.3±3.8	<.01

Abbreviations: PWS, Preferred Walking Speed; 130%PWS, 130% of the Preferred Walking Speed; step length-A, step length of Affected limb; step length-U, step length of Unaffected limb; SST-A, Single Stance Time of Affected limb; SST-U, Single Stance Time of Unaffected limb; TDST, Terminal Double Stance Time; IDST, Initial Double Stance Time; SSTP-A, Single Stance Time of Affected limb as a Percentage of the gait cycle; SSTP-U, Single Stance Time of Unaffected limb as a Percentage of the gait cycle; TDSTP, Terminal Double Stance Time as a Percentage of the gait cycle; IDSTP, Initial Double Stance Time as a Percentage of the gait cycle.

**Table3:** Number of subjects showing increase, decrease or no change in each time-distance parameter at 130% PWS condition compared with PWS condition

Parameter	PWS<130% PWS (n)	PWS>130% PWS (n)	PWS=130% PWS (n)	Percentage of cases that differed from the overall trend (%)
cadence	42	1	0	2.3%
stride length	43	0	0	0.0%
step length-A	40	3	0	7.0%
step length-U	42	1	0	2.3%
SST-A	14	29	0	32.6%
SST-U	15	28	0	34.9%
TDST	5	38	0	11.6%
IDST	5	37	1	14.0%
SSTP-A	35	8	0	18.6%
SSTP-U	35	8	0	18.6%
TDSTP	10	33	0	23.3%
IDSTP	11	32	0	25.6%

Abbreviations: PWS, Preferred Walking Speed; 130%PWS, 130% of the Preferred Walking Speed; step length-A, step length of Affected limb; step length-U, step length of Unaffected limb; SST-A, Single Stance Time of Affected limb; SST-U, Single Stance Time of Unaffected limb; TDST, Terminal Double Stance Time; IDST, Initial Double Stance Time; SSTP-A, Single Stance Time of Affected limb as a Percentage of the gait cycle; SSTP-U, Single Stance Time of Unaffected limb as a Percentage of the gait cycle; TDSTP, Terminal Double Stance Time as a Percentage of the gait cycle; IDSTP, Initial Double Stance Time as a Percentage of the gait cycle. Gray scale denotes the number of subjects showing the overall trend for each parameter.

## Supplementary Material

PDF-Japanese
