# Supplementary material for: Effects of change in walking speed on time-distance parameters in post-stroke hemiplegic gait
Source: Fujita Med J. 2022 Jan 25;8(4):121–6. doi: 10.20407/fmj.2021-016 (PMC9673078; doi:10.20407/fmj.2021-016)
Supplement: Supplementary file 1 — PDF-Japanese [file fmj-8-121_s001.pdf]

論文タイトル：歩行速度変化が脳卒中片麻痺歩行の時間距離因子に与える影響
ランニングタイトル：快適歩行速度条件と速歩条件での比較

Authors:

Ken Tomida (RPT, MS)[1,2)]
Toshio Teranishi (RPT, PhD)[3)]
Misaki Takai (RPT)[2)]
Kenji Kawakami (RPT, MS)[2)]
Kei Ohtsuka (RPT, PhD)[3)]
Hiroki Ogawa (RPT)[2)]
Akira Suzuki (RPT)[2)]
Shigeru Sonoda (MD, PhD)[2,4)]

Affiliations:

1）Graduate School of Health Sciences, Fujita Health University, Toyoake, Aichi, Japan
2）Fujita Health University Nanakuri Memorial Hospital, Tsu, Mie, Japan
3）Faculty of Rehabilitation, School of Health Sciences, Fujita Health University, Toyoake, Aichi, Japan
4）Department of Rehabilitation Medicine II, School of Medicine, Fujita Health University, Tsu, Mie, Japan

Article types: Original Article

Corresponding author:

Name: Kei Ohtsuka
Affiliation:Faculty of Rehabilitation, School of Health Science, Fujita Health University
Zip code: 470-1192
Address: 1-98, Dengakukekubo, Kutsukakecho, Toyoake, Aichi 470-1192, Japan.
Telephone: 0562-93-2000
E-mail: ohtsuka@fujita-hu.ac.jp

アブストラクト

目的:

脳卒中患者の歩行能力を経時的に評価することは重要である。計測装置を用いた歩行の定量評価では、計測速度条件を計測時点での快適歩行速度や最大歩行速度を基準に設定することが多い。しかし、回復段階では歩行速度が増加することが多いため、経過を比較する際に速度変化が歩行に与える影響を理解しておく必要がある。歩行速度変化が脳卒中患者の歩行に与える影響を報告した先行研究はいくつかあるが、時間距離因子について言及した報告は、症例数が少ないため一般化しにくい。

そこで我々は、脳卒中片麻痺患者 43 例を対象に、快適歩行速度（Preferred Walking Speed、PWS）と PWS の 1.3 倍の速度（以下、130%PWS）でトレッドミル歩行を計測し、歩行速度変化が時間距離因子に与える影響を分析したので報告する。

方法:

対象は初発脳卒中片麻痺患者とし、監視レベルにてトレッドミル歩行が可能な 43 名とした。三次元動作解析装置を用い、PWS と 130%PWS の 2 条件でトレッドミル歩行を計測した。主要評価項目は時間距離因子とし、PWS と 130%PWS の 2 条件間で比較した。

結果:

cadence と stride length、麻痺側・非麻痺側歩幅は 130%PWS で有意に増加した。実時間である麻痺側・非麻痺側単脚支持時間、後期・前期両脚支持時間は 130%PWS で有意に減少した。相対時間である麻痺側・非麻痺側単脚支持相対時間は 130%PWS で有意に増加し、後期・前期両脚支持相対時間は、130%PWS で有意に減少した。

結論:

初発脳卒中片麻痺患者のトレッドミル歩行において、歩行速度変化による時間距離因子への影響が明らかとなった。本研究の結果が異なる速度条件で計測された時間距離因子を解釈する際の一助になれば幸いである。

キーワード: Stroke; Time-distance parameters; Walking speed

目的

脳卒中片麻痺患者の歩行能力の改善は、脳卒中患者のリハビリテーションにおいて重要な目標の 1 つである。経時的に、その効果である歩行能力の変化を査定するためには、適切な歩行能力の評価手法が必要である。これまで、歩行能力評価には、Functional Ambulation Categories や Functional Independence Measure（FIM）、Gait Ability Assessment for hemiplegics（GAA）を用いた歩行自立度[1-3]や 5m や 10m 歩行時間測定による歩行速度[4]などがあり、運動学・運動力学的評価としては 3 次元動作解析装置や床反力計を用いた計測[5,6]がある。これら計測装置は、歩行中の時間距離因子や関節角度、関節モーメント等の定量評価が可能で、有効な評価手段である。

これら計測装置を用いて歩行を評価する際、歩行速度条件は計測時点での快適歩行速度や最大歩行速度を基準に設定することが多い。しかし脳卒中片麻痺患者の回復期の場合、一般的には経過に伴い脳卒中片麻痺患者の歩行速度は増加することが多く[7]、1 回目のテストと 2 回目のテスト時の快適等の歩行条件は速度が異なる可能性が高い。それ故、評価結果を解釈する際、計測時期による歩行速度変化の影響を理解しておくことは必須と考える。

健常者を対象に歩行速度変化が歩行時間距離因子に与える影響を調査した先行研究[8-13]は多数みられる。脳卒中片麻痺患者では、速度別の時間距離因子について、基本情報を一致させた健常者と比較した多数例での調査[14]はあるものの、速度変化そのものが脳卒中片麻痺患者の歩行時間距離因子に与える影響を調査した報告は多くはない。脳卒中片麻痺患者を対象とした先行研究を紐解くと、時間距離因子[15-17]、関節角度[15,16]、筋出力[17]の影響について報告しているものがある。臨床的に用いられやすい時間距離因子に着目すると、少数例での検討[16,17]や stride length、step length などの距離因子と cadence などの代表的な時間因子に対する速度変化の影響を報告したもの[15]はあるものの、多数例で時間距離因子を細分化し網羅的に調査した報告は少ない。

そこで我々は、脳卒中片麻痺患者 43 例を対象に、快適歩行速度条件と快適歩行速度の 1.3 倍の 2 つの速度条件でトレッドミル歩行を計測し、歩行速度変化が時間距離因子に与える影響を分析した。距離因子に加え、時間因子では cadence、実時間因子、相対時間因子について網羅的に分析したので報告する。

対象と方法

研究デザイン

Retrospective cohort study

対象

　対象は、当院回復期リハビリテーション病棟に入院した初発脳卒中片麻痺患者とし、入院後 6 週時に、ankle-foot orthosis（AFO）を装着して監視レベルにてトレッドミル歩行が可能な 43 名とした。患者基本情報として、年齢、性別、麻痺側、発症後期間、FIM-walk score（以下、FIM-walk）[2]、麻痺側下肢運動機能として Stroke Impairment Assessment Set（SIAS） 下肢運動項目合計（以下、SIAS-L/E）[18] を評価した（table.1）。本研究は藤田医科大学医学研究倫理審査委員会にて承認（HM19-254）を得ている。

計測

　トレッドミル歩行計測は三次元動作解析装置 Kinema Tracer®(Kissei Comtec Co., Ltd., Matsumoto, Japan)を使用し、トレッドミル歩行を計測した。全例手すりを使用し、装具は理学療法における歩行練習で使用する設定とした。計測速度は、平地快適歩行速度（Preferred Walking Speed、PWS）と平地快適歩行速度の 1.3 倍（以下、130%PWS）とし、転倒予防のために safety harness を使用するが、体重の免荷は行わなかった。

　トレッドミル歩行計測は、先行研究[5]に従った方法で計測した。マーカーの貼付位置は、両側の肩峰、股関節、膝関節、外果、つま先の 10 箇所とし、サンプリング周波数は 60Hz にて計測時間は 20 秒とした。

　計測速度の基礎となる PWS を決定するために、対象者の好ましい速度で平地の 10m 歩行時間を 3 回計測し、その平均値を PWS と定義した。また、トレッドミル歩行計測前には対象者がトレッドミル歩行に慣れるよう十分に練習を行った。

評価項目

　主要評価項目は、トレッドミル歩行計測時の時間距離因子とした。具体的には、歩行速度（walking speed）、重複歩距離（stride length）、ケーデンス（cadence）、麻痺側の歩幅（step length of Affected limb、step length-A）、非麻痺側の歩幅（step length of Unaffected limb、step length-U）、麻痺側の単脚支持時間（Single Stance Time of Affected limb、SST-A）、非麻痺側の単脚支持時間（Single Stance Time of Unaffected limb、SST-U）、麻痺側から荷重が離れる際の両脚支持時間（Terminal Double Stance Time、TDST）、麻痺側に加重する際の両脚支持時間（Initial Double Stance Time、IDST）、SST-A の相対時間（Single Stance Time of Affected limb as a Percentage of the gait cycle、SSTP-A）、SST-U の相対時間（Single Stance Time of Unaffected limb as a Percentage of the gait cycle、SSTP-U）、TDST の相対時間（Terminal Double Stance Time as a Percentage of the gait

cycle、TDSTP)、IDST の相対時間（Initial Double Stance Time as a Percentage of the gait cycle、IDSTP）とした。

統計学的処理

統計学的解析には SPSS Statistics 19 (International Business Machines Corp., Armonk, NY, USA)を使用した。時間距離因子における 2 つの速度条件間の比較には、対応のある t 検定を用い、危険率 5%をもって有意とした。

結果

歩行速度は PWS が 2.0±0.7km/h、130%PWS が 2.6±0.9km/h であった。cadence と距離因子（table 2、 figure 1）である stride length、step length-A、step length-U は 130%PWS で有意に増加した。時間因子のうち、実時間である SST-A、SST-U、TDST、IDST は 130%PWS で有意に減少した (table 2、 figure 2)。相対時間である SSTP-A と SSTP-U は 130%PWS で有意に増加し、TDSTP と IDSTP は、130%PWS で有意に減少した（table 2、 figure 3)。

各時間距離因子において 130%PWS で増加した人数と減少した人数を table3 に示した。全体の傾向とは異なる結果を示した症例数（table 3）は、cadence、stride length、step length-A、 step length-U、TDST、IDST、SSTP-A、SSTP-U、TDSTP、IDSTP は 30%未満であったのに対し、SST-A、SST-U は 32.6%、34.9%とやや多い傾向にあった。

考察

本研究では、脳卒中片麻痺患者を対象として速度変化に伴う時間距離因子の変化を検討した。歩行速度の増加に伴い、距離因子は全て有意に増加し、実時間因子は全て有意に減少した。相対時間因子では、SSTP-A と SSTP-U が有意に増加し、TDSTP と IDSTP が有意に減少した。cadence は有意に増加した。

Murray ら [8] は健常者を対象とし、free speed と fast speed での歩行を分析している。時間距離因子では、1 歩行周期時間、立脚期時間、遊脚期時間、両脚支持時間、立脚相対時間、両脚相対時間が減少し、遊脚相対時間、stride length、cadence が増加したと報告している。また、Liu Y ら [12] も速度増加に伴い、1 歩行周期時間、立脚相対時間、両脚相対時間が減少し、遊脚相対時間、stride length、cadence が増加したと報告している。

Tyrell ら [16] は脳卒中患者 20 例を対象とし、速度負荷が歩行に与える影響を時間距離因子と運動学的因子から分析している。時間距離因子は速度増加に伴い、麻痺側と非麻痺側の歩幅、麻痺側と非麻痺側の単脚支持相対時間が増加し、麻痺側と非麻痺側の両脚支持相対時間が減少すると報告している。Lamontagne ら [17]

も脳卒中患者 12 例を対象とし、同様の結果を報告している。これら先行研究を概観すると、速度負荷が歩行の時間距離因子に与える影響は、健常者も脳卒中片麻痺患者でも同傾向であるのがわかる。一方、脳卒中患者は個人間変動が大きいことが知られている[19]。Tyrell ら[16] は Limitations の 1 つとして、サンプルサイズが小さいことを挙げている。脳卒中片麻痺患者 43 名で分析した本研究の結果は、先行研究と同様の傾向であったことから、個人変動が大きいとされる脳卒中患者であっても、本結果を一般化することができると考えられる。

速度増加に対応するためには、cadence と stride length を増加させる必要があることは広く知られており[8,9,12,13,15,]、cadence の増加は 1 歩行周期時間の減少と同義である。脳卒中片麻痺患者の各期での実時間の減少は、速度負荷に対応するための時間的戦略と考察する。

一方、相対時間では麻痺側、非麻痺側を問わず単脚支持相対時間が増加し、両脚支持相対時間が減少した。Murray ら[8] は健常者を対象に速度増加が歩行に対する影響を分析している。時間因子の考察の中で、速度負荷に伴う遊脚時間の減少量が他相の減少量と比較して少ないのは、より短い時間でより遠くに下肢を振り出すことで歩幅を確保する必要があるためであると考察している。また、脳卒中患者の歩行速度増加の要因として、立脚後期での push-off（足関節底屈）と遊脚初期での pull-off（股関節屈曲）のモーメント増加や角速度の増加による推進力の生成や素早い下肢の振り出しが重要[20,21] であり、この円滑な push-off と pull-off が両脚支持時間の減少を支持していると考えられる。つまり、遊脚時間は、速度負荷の対応として歩幅を増加させた結果、素早い振り出しや推進力を得るために減少した両脚支持時間よりも実時間の減少比が少なくなったため、麻痺側・非麻痺側ともに単脚支持相対時間が増加し、両脚支持相対時間が減少したと考察した。

症例ごとの結果に目を向けると、単脚支持時間は麻痺側、非麻痺側ともに全体の傾向と異なる結果を呈する症例の割合がやや多い結果となった。Beaman ら[22] は速度負荷に対する戦略は、一律ではないことを報告している。また、脳卒中患者は個人間変動が大きいことが知られている[19] ことから、麻痺側または非麻痺側の機能や対応の仕方に個体差があったことが、要因となったと推察した。一方、速度負荷に対し非典型な傾向を示す原因を明らかにすることは、歩行能力評価や歩行練習プログラムの立案に有用であると考えられ、今後の課題としたい。

計測装置を用いて歩行を評価する際、歩行速度条件は計測時点での快適歩行速度や最大歩行速度を基準に設定することが多い。本研究の結果が異なる速度条件で計測された時間距離因子を解釈する際の一助になれば幸いである。

研究限界

本研究では、計測時に手すりを使用させていたことから、手すりの使用が速度変化と時間距離因子変化に与える影響が、本研究の交絡因子となっている可能性が考えられる。また、本研究の対象は装具を使用した症例となっていることにも留意が必要である。

結論

初発脳卒中片麻痺患者 43 例を対象に、PWS と 130%PWS の 2 条件で 3 次元動作解析装置によるトレッドミル歩行計測を行なった。対象は装具と手すりの使用を許可し、免荷をせずに転倒予防のための safety harness を使用して計測した。装具は理学療法中の歩行練習で使用する設定とした。時間距離因子を主要評価項目として、PWS と 130%PWS の 2 条件間で比較した。

cadence、stride length、step length-A、 step length-U は 130%PWS で有意に増加した。時間因子のうち、実時間である SST-A、SST-U、TDST、IDST は 130%PWS で有意に減少した。相対時間である SSTP-A と SSTP-U は 130%PWS で有意に増加し、TDSTP と IDSTP は、130%PWS で有意に減少した。これらの傾向は、先行研究で健常者を対象に検討された報告と同様の傾向を示した。一方、SST-A、SST-U については、全体の傾向と異なる結果を示す症例が一定数いることがわかった。

謝辞

本研究を進めるにあたり、多大なるご指導を頂きました多くの先生方に深くお礼申し上げます。また、研究をサポートして頂きました藤田医科大学七栗記念病院のリハビリテーション部の皆様、データ解析にご協力頂きました市原峰子氏に深くお礼申し上げます。

利益相反

本論文に関して、開示すべき利益相反関連事項はない。

引用文献

1. Holden MK, Gill KM, Magliozzi MR, Nathan J, Piehl-Baker L. Clinical gait assessment in the neurologically impaired. Reliability and meaningfulness. Phys Ther 1984; 64: 35 - 40.

2. Data management service of the Uniform Data System for Medical Rehabilitation, Center for Functional Assessment Research. Guide for use of the uniform data set for medical rehabilitation. Ver.3.0. Buffalo, NY: State

University of New York at Buffalo; 1990.

3. Tomida K, Tanino G, Sonoda S, Hirano S, Itoh N, Saitoh E, Kagaya H, Suzuki A, Kawakami K, Miyajima T, Takai M. Development of Gait Ability Assessment for hemiplegics (GAA) and verification of inter-rater reliability and validity. Japanese Journal of Comprehensive Rehabilitation Science 2021; 12: 19-26.

4. Collen FM, Wade DT, Bradshaw CM. Mobility after stroke: reliability of measures of impairment and disability. Int Disabil Stud 1990; 12: 6-9.

5. Mukaino M, Ohtsuka K, Tanikawa H, Matsuda F, Yamada J, Itoh N, Saitoh E. Clinical-oriented Three-dimensional Gait Analysis Method for Evaluating Gait Disorder. J Vis Exp 2018; 133: 57063

6. Morita S, Yamamoto H, Furuya K. Gait analysis of hemiplegic patients by measurement of ground reaction force. Scand J Rehabil Med 1995; 27: 37-42.

7. Yamada S, Tomida K, Tanino G, Suzuki A, Kawakami K, Kubota S, Yanohara R, Katoh Y, Wada Y, Teranishi T, Orand A, Tomita Y, Sonoda S. How effective is the early fast treadmill gait speed training for stroke patients at the 2nd week after admission: comparison with comfortable gait speed at the 6th week. J Phys Ther Sci 2015; 27: 1247-50.

8. Murray MP, Kory RC, Clarkson BH, Sepic SB. Comparison of free and fast speed walking patterns of normal men. Am J Phys Med 1966; 45: 8-23.

9. Murray MP, Mollinger LA, Gardner GM, Sepic SB. Kinematic and EMG patterns during slow, free, and fast walking. J Orthop Res 1984; 2: 272-80.

10. Jordan K, Challis JH, Newell KM. Walking speed influences on gait cycle variability. Gait Posture 2007; 26: 128-34.

11. Kwon JW, Son SM, Lee NK. Changes of kinematic parameters of lower extremities with gait speed: a 3D motion analysis study. J Phys Ther Sci 2015; 27: 477-79.

12. Liu Y, Lu K, Yan S, Sun M, Lester DK, Zhang K. Gait phase varies over velocities. Gait Posture 2014; 39: 756-60.

13. Larsson LE, Odenrick P, Sandlund B, Weitz P, Oberg PA. The phases of the stride length and their interaction in human gait. Scand J Rehabil Med 1980; 12: 107-12.

14. Wang Y, Mukaino M, Ohtsuka K, Otaka Y, Tanikawa H, Matsuda F, Tsuchiyama K, Yamada J, Saitoh E. Gait characteristics of post-stroke hemiparetic patients with different walking speeds. Int J Rehabil Res 2020; 43: 69-75.

15. Hutin E, Pradon D, Barbier F, Bussel B, Gracies JM, Roche N. Walking velocity and lower limb coordination in hemiparesis. Gait Posture 2012; 36: 205-11.

16. Tyrell CM, Roos MA, Rudolph KS, Reisman DS. Influence of systematic increases in treadmill walking speed on gait kinematics after stroke. Phys Ther 2011; 91: 392-403.

17. Lamontagne A, Fung J. Faster is better: implications for speed-intensive gait training after stroke. Stroke 2004; 35: 2543-48.

18. Chino N, Sonoda S, Domen K, Saitoh E, Kimura A. Stroke impairment assessment set (SIAS): a new evaluation instrument for stroke patients. The Japanese Journal of Rehabilitation Medicine 1994; 31: 119 - 25.

19. Woolley SM. Characteristics of gait in hemiplegia. Top Stroke Rehabil 2001; 7: 1-18.

20. Nadeau S, Gravel D, Arsenault AB, Bourbonnais D. Plantarflexor weakness as a limiting factor of gait speed in stroke subjects and the compensating role of hip flexors. Clin Biomech (Bristol, Avon) 1999; 14: 125-35.

21. Olney SJ, Griffin MP, McBride ID. Temporal, kinematic, and kinetic variables

related to gait speed in subjects with hemiplegia: a regression approach. Phys Ther 1994; 74: 872-85.

22. Beaman CB, Peterson CL, Neptune RR, Kautz SA. Differences in self-selected and fastest-comfortable walking in post-stroke hemiparetic persons. Gait Posture 2010; 31: 311-316.
